# Supplementary material for: Effect of Household Type on the Prevalence of Climacteric Syndrome among Middle-Aged Men
Source: Healthcare (Basel). 2023 Oct 5;11(19):2684. doi: 10.3390/healthcare11192684 (PMC10572107; doi:10.3390/healthcare11192684)
Supplement: Supplementary file 1 [file healthcare-11-02684-s001.zip › healthcare-2624296-supplementary.pdf]

Effect of Household Type on the Prevalence of Climacteric Syndrome among Middle-Aged Men

Table S1. General characteristics associated with climacteric syndrome based on aging males' symptoms subscale.

Variable		Psychological subscale		Somatic subscale		Sexual subscale	
		n (%)	*p*	*n* (%)	*p*	*n* (%)	*p*
Age	Less than 50	370(86.2)	0.328	371(86.5)	0.005**	400(93.2)	<0.001***
	50 or more	680(88.2)		706(91.6)		752(97.5)	
Education level	University	846(86.6)	0.046*	866(88.6)	0.008**	963(95.8)	0.467
	High school	204(91.5)		211(94.6)		216(96.9)	
Occupation	White-collar	829(87.2)	0.501	846(89.0)	0.077	911(95.8)	0.476
	Blue-collar	221(88.8)		231(92.8)		241(96.8)	
Income level	High	519(86.2)	0.176	535(88.9)	0.313	575(95.5)	0.390
	Low	531(88.8)		542(90.6)		577(96.5)	
Smoking status	Non-smoker	603(87.1)	0.659	605(87.4)	0.002**	664(96.0)	0.924
	Smoker	447(88.0)		472(92.9)		488(96.1)	
Drinking behavior	Low-risk	829(87.1)	0.389	849(89.2)	0.203	910(95.6)	0.154
	High-risk	221(89.1)		228(91.9)		242(97.6)	
Physical activity	Adequate	199(89.2)	0.385	193(86.5)	0.081	221(99.1)	0.009**
	Inadequate	851(87.1)		884(90.5)		931(95.3)	
Sleep duration	Sufficient	536(89.2)	0.077	534(88.9)	0.304	579(96.3)	0.548
	Insufficient	514(85.8)		543(90.7)		573(95.7)	
BMI	Normal	596(86.8)	0.366	613(89.2)	0.491	666(95.5)	0.295
	Obesity	454(88.8)		464(90.4)		496(96.7)	
Household type	Multi-person	502(83.7)	<0.001***	519(86.5)	<0.001***	572(95.3)	0.239
	Single-person	548(91.3)		558(93.0)		580(96.7)	

* $p < 0.05$, ** $p < 0.01$, *** $p < 0.001$.

Table S2. Dietary factor associated with climacteric syndrome based on aging males' symptoms subscale.

Variables		Psychological subscale		Somatic subscale		Sexual subscale	
		n (%)	*p*	*n* (%)	*p*	*n* (%)	*p*
Breakfast frequency	Five or more times a week	320(81.0)	<0.001***	338(85.6)	0.001**	377(95.4)	0.490
	Four or fewer times a week	730(90.7)		739(91.8)		775(96.3)	
Shared breakfast	Together	274(82.8)	0.002**	287(86.7)	0.032*	314(94.9)	0.215
	Alone	776(89.3)		790(90.9)		838(96.4)	
Lunch frequency	Five or more times a week	900(87.9)	0.324	919(89.7)	0.991	984(96.1)	0.689
	Four or fewer times a week	150(85.2)		158(89.8)		168(95.5)	
Shared lunch	Together	734(87.2)	0.600	764(90.7)	0.084	812(96.4)	0.236
	Alone	316(88.3)		313(87.4)		340(95.0)	
Dinner frequency	Five or more times a week	795(85.0)	<0.001***	825(88.2)	0.001**	889(95.1)	0.002**
	Four or fewer times a week	255(96.2)		252(95.1)		263(99.2)	
Shared dinner	Together	618(86.0)	0.048*	630(87.6)	0.003**	688(95.7)	0.501
	Alone	432(89.8)		447(92.9)		464(96.5)	
Eating out	Two or fewer times a week	524(85.1)	0.009**	539(87.5)	0.008**	588(95.5)	0.322
	Three or more times a week	526(90.1)		538(92.1)		564(96.6)	

*$p < 0.05$, **$p < 0.01$, ***$p < 0.001$.
